# Study designs

**DOI:** 10.4103/0974-7788.64406

**Published:** 2010

**Authors:** Shraddha Parab, Supriya Bhalerao

**Affiliations:** *Department of Clinical Pharmacology, Seth GS Medical College and KEM Hospital, Parel, Mumbai - 400 012, India*; 1 *Department of Clinical Pharmacology, TNMC and BYL Nair Hospital, Mumbai Central, Mumbai - 400 008, India*

In the last issue, we discussed "sample size", one of the crucial aspects when planning a clinical study. This article discusses another statistically important issue, Study designs. Study design is a process wherein the trial methodology and statistical analysis are organized to ensure that the null hypothesis is either accepted or rejected and the conclusions arrived at reflect the truth.

The design of any study is more important than analyzing its results, as a poorly designed study can never be recovered, whereas a poorly analyzed study can be reanalyzed to reach a meaningful conclusion.[1] Rather, the design of the study decides how the data generated can be best analyzed. The scientific integrity of the study and the credibility of the data from the study thus substantially depend on the study design.

The various aspects of clinical research can be broadly divided into two types, viz., observational and experimental. The basic difference between these two types is that the earlier does not involve any intervention (drug treatment/therapeutic procedures/diagnostic tools), whereas in an experimental study, the investigator administers an intervention to patients and the effect of this intervention on the course of events is documented. Let us see the different designs which are commonly used to conduct these two types of researches.

## DESCRIPTIVE STUDY

This is the first foray into research. These studies describe the frequency, natural history and determinants of a factor/disease. It is a study to identify patterns or trends in a situation, but not the cause and effect (causal) linkages among its different elements, e.g. a study to assess the predominant *prakriti* in hypertensive patients only helps in determining the predominant *prakriti* in these patients, it does not establish a linkage that a specific *prakriti* is a causative factor for hypertension. Types of descriptive studies are prevalence surveys, case series, surveillance data and analysis of routinely collected data, etc.

### Case series and case reports

A case report is a descriptive study of a single individual, whereas case series is a study of a small group. In these studies, the possibility of an association between an observed effect and a specific environmental exposure is studied based on detailed clinical evaluations and histories of the individual(s).

They are most likely to be useful when the disease is uncommon and caused exclusively by a single kind of exposure (e.g. vinyl chloride and angiosarcoma or diethylstilbestrol (DES) and clear-cell carcinoma of the vagina).[2] Case reports (or case series) may be first to provide clues in identifying a new disease or adverse health effect from an exposure.

## ANALYTICAL STUDY

These studies are generally (although not always) used to test one or more specific hypotheses, typically whether an exposure is a risk factor for a disease or an intervention is effective in preventing or curing disease (or any other occurrence or condition of interest). Of course, data obtained in an analytic study can also be explored in a descriptive mode, and data obtained in a descriptive study can be analyzed to test hypotheses, making it analytical. In short, these studies are designed to examine etiology and causal associations. Types of analytical studies are cross sectional, case-control, cohort (retrospective and prospective) and ecological.

### Cross sectional

Cross-sectional study is also known as a prevalence study. It measures the cause and effect at the same time, but does not tell us the relationship, i.e. which one is the cause and which one is the effect. This is the commonest study design used in general practice and research, in general. These studies are relatively easy to do, inexpensive and can be carried out in a short time frame.

### Case-control

**Figure 1:**
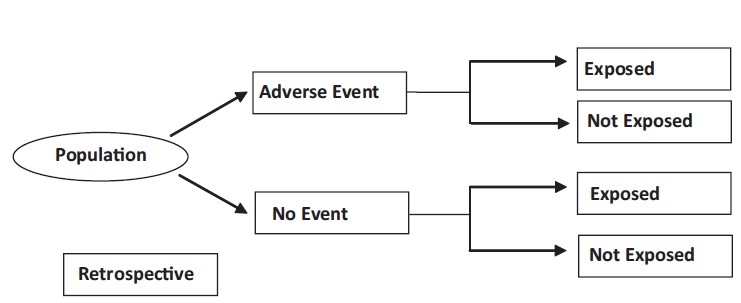
Case-Control design

In studies using this particular design, patients who already have a certain condition (cases) are compared (e.g. diabetic patients with hospitalization) with people who do not have that condition (controls) (e.g. diabetic patients without hospitalization). The researcher goes through the past records of these subjects (both cases and controls) to find out whether the development of the condition only in one group of patients is due to presence of some causative factor (exposure). Thus, in a typical case-control study, the data collection is mainly retrospective (backward in time) [Figure 1].

These studies are less reliable than either randomized controlled trials or cohort studies. A major drawback to case-control studies is that one cannot measure the risk of developing a particular outcome because of an exposure. Additionally, in these studies one has to mainly rely on the memory of patients to identify what in the past might have caused their current disease, which is most often of long latency. This might induce a bias while analyzing the results, which is known as "recall bias". Because human memory is frequently imprecise, recall bias is commonly believed to be "pervasive in case-control studies."[3]

The presence of disease affects both the patient's perception of the causes and his search for possible exposure to a hypothesized risk factor. Therefore, the recall of remote exposures in case-control studies is commonly presumed to be differential among study subjects depending on their disease status.[4] Data, even about irrelevant exposures, are often remembered better by cases or/and underreported by controls.[5] This trend in exposure recall tends to inflate the risk estimate in case-control studies. Also, recalling the exact timing of exposure, which is often important in determining temporality of an association and in estimating induction period of a disease, can be differential among exposed cases and exposed controls.

Despite the fact that recall bias is a major limitation of case-control studies, a number of methodological strategies documented in the literature can minimize the recall bias.[6]

The advantages of case-control studies are that they can be done quickly and are very efficient for conditions/diseases with rare outcomes.[7]

### Cohort (Longitudinal studies)

A cohort study begins with a group of subjects with some causative factor (e.g. daily intake of *Virrudha ahar*) but free of the condition of interest (e.g. skin diseases). All the subjects are followed up and observed for the occurrence of the condition of interest.

**Figure 2:**
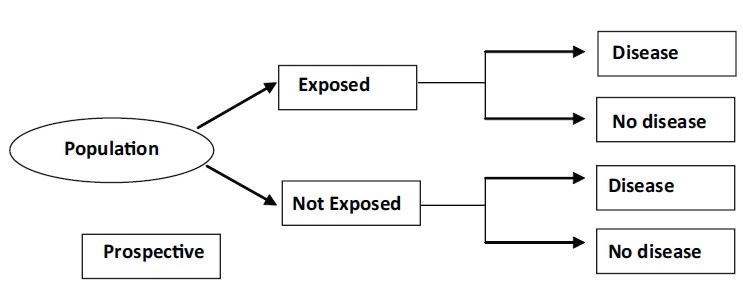
Cohort (Longitudinal studies) design

In contrast to the case-control study, a cohort study is usually prospective (forward in time). It provides the best information about the cause of disease plus the most direct measurement of the risk of developing a particular outcome due to exposure [Figure 2].

These studies, however, require a large number of subjects and a long period of follow up to assess whether the event of interest has occurred, due to which these studies are very expensive to conduct. The main drawback of these studies due to long follow up is that there are high chances of subjects getting lost to follow up.[7] If in the two groups, the degree of such losses is substantially different, it can lead to bias and false positive results.

### Correlational studies

These studies (sometimes called ecologic studies) explore the statistical connection between disease in different population groups and estimated exposures in groups rather than individuals. For example, they may correlate death rates by country with estimates of exposure, such as factory emissions in a given geographic area, proximity to waste sites, or air or water pollution levels. The geographical information system (GIS) is a very useful new tool that improves the ability of ecologic studies to be able to determine a link between health data and a source of environmental exposure.

## CONTROLLED STUDIES

These studies have control groups (i.e. comparator that can either be a standard drug or placebo). Controlled trials can be clinical trials (unit of randomization is an individual) or community trials (unit of randomization is a community or cluster).

### Nonrandomized controlled

This is an experimental study in which people are allocated to different interventions using methods that are not random. In these studies, allocation to different groups is done arbitrarily. This kind of study design may sometimes overestimate the advantages of one treatment over other.

### Randomized controlled

Randomized controlled trials (RCTs) are considered the "gold standard" in medical research since they offer the best answers about the effectiveness of different therapies or interventions.

**Figure 3:**
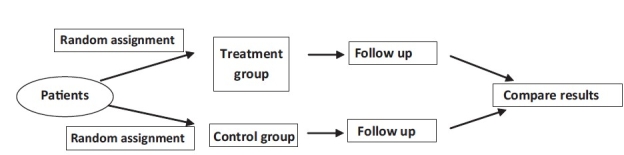
Randomised clinical trial

The important aspect of this study design is that the patients are randomly assigned to the study all groups that help in avoiding bias in patient allocation-to-treatment that a physician might be subject to [Figure 3]. It also increases the probability that the differences between the groups can be attributed only to the treatment(s) under study.

There are certain types of questions where randomized controlled studies cannot be done for ethical reasons, for instance, if patients are asked to undertake harmful experiences (like smoking) or denied any treatment beyond a placebo when there are known effective treatments.

There are different types of randomized studies as follows.[8]

#### Parallel

**Figure 4:**
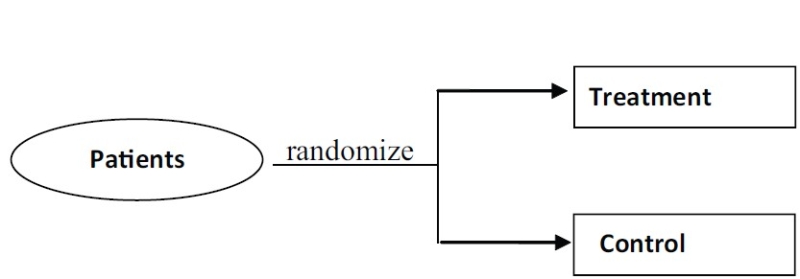
Parallel design

In parallel studies, treatment and controls are allocated to different individuals. This is unlike a crossover study where at first one group receives treatment A, followed by treatment B later, while the other group receives treatment B followed by treatment A [Figure 4].

In case of Ayurvedic studies, an extension of this design known as "add-on" design is useful, where one group receives standard treatment, while the other group receives standard treatment along with Ayurvedic treatment. Using these studies, comparison of relative or absolute efficacy can be obtained in a short period. However, these studies generally require large number of patients for the analysis.

#### Crossover

**Figure 5:**
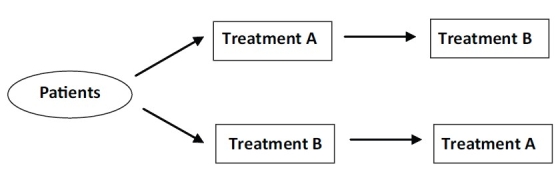
Cross over design

In these types of studies each patient serves as his own control. Each patient gets both drugs; the order in which the patient gets each drug is randomized [Figure 5]. Generally, it requires a smaller sample size.[9]

#### Assumptions

The effects of intervention during the first period do not carry over into the second period.

Internal factors (e.g. disease severity) and external factors (e.g. season), which can affect the efficacy of the drug/s, are constant over time.

Ideally, the patient's disease condition should return to its baseline state after discontinuation of the first treatment.

In case of Ayurvedic studies, this design can prove useful as each patient serves as his own control and this way the individualistic approach of Ayurveda gets conserved even in clinical studies. However, at the same time it is difficult to implement as the "wash out period" (duration between two treatments to wash out the effect of the first so that it does not get carry over) cannot be defined in view of unavailability of pharmacokinetic data of Ayurvedic treatments.

#### Factorial

**Figure 6:**
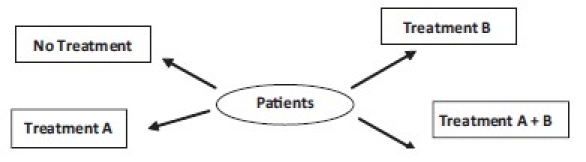
Factorial design

Studies involving two or more factors while randomizing are called factorial designs [Figure 6]. A factor is simply a categorical variable (e.g. age and *prakriti*) with two or more values, referred to as levels.

Factorial design permits researchers to investigate the joint effect of two or more factors on a dependent variable (e.g. weight). The factorial design also facilitates the study of interactions, illuminating the effects of different conditions of the experiment on identifiable subgroups of subjects participating in the experiment.

#### Cluster

It is a type of randomized controlled trial wherein groups of participants (as opposed to individual participants) are randomized. Cluster randomized controlled trials are also known as cluster randomized trials, group randomized trials, and place randomized trials.

Advantages of cluster randomized controlled trials over individually randomized controlled trials include the ability to study interventions that cannot be directed toward selected individuals (e.g. a radio show about lifestyle changes) and the ability to control for "contamination" across individuals (e.g. one individual's change in behavior may influence another individual to do so too). Disadvantages compared with individually randomized controlled trials include greater complexity in design and analysis and a requirement for more participants to obtain the same statistical power.

### Quasi-randomized

In these studies, participant allocation is done using schemes such as date of birth (odd or even), number of the hospital record, date at which they are invited to participate in the study (odd or even), or alternatively into different study groups.

A quasi-randomized trial uses quasi-random method of allocating participants to different interventions. There is a greater risk of selection bias in quasi-random trials where allocation is not adequately concealed compared with randomized controlled trials with adequate allocation concealment.
